# Mutant *C. elegans* p53 Together with Gain-of-Function GLP-1/Notch Decreases UVC-Damage-Induced Germline Cell Death but Increases PARP Inhibitor-Induced Germline Cell Death

**DOI:** 10.3390/cancers14194929

**Published:** 2022-10-08

**Authors:** Jorge Canar, Prima Manandhar-Sasaki, Jill Bargonetti

**Affiliations:** 1 Department of Biological Sciences, Hunter College, City University of New York, New York, NY 10065, USA; 2 Macaulay Honors College at Hunter College, City University of New York, New York, NY 10065, USA; 3 The Graduate Center, Departments of Biology and Biochemistry, City University of New York, New York, NY 10016, USA; 4 Department of Cell and Developmental Biology, Weill Cornell Medical College, New York, NY 10065, USA

**Keywords:** p53, CEP-1, cancer, DNA damage, PARP inhibitor, lifespan

## Abstract

**Simple Summary:**

The tumor suppressor gene *TP53* is conserved from nematode to human. In humans the *TP53* gene is found mutated in a majority of cancers and therefore, the p53-induced cell death pathway is dysfunctional. As such, it is of the utmost importance to determine mechanisms and models to test for ways to induce p53-independent cancer cell death. The small, transparent, nematode *C. elegans* is a whole animal with a germline stem cell tumor model that presents such an opportunity. We used this model with a well-studied p53 mutant that increases germline tumor size to test for ways to induce p53-independent cell death. Herein, we report that two p53-independent death inducers, a nucleoside analogue and a PARP inhibitor, are capable of inducing *C. elegans* germline tumor cell death. This suggests new targeted drugs can be tested in this model for p53-independent cancer cell killing.

**Abstract:**

The *TP53* gene is mutated in over 50% of human cancers, and the *C. elegans* *p53-1* *(cep-1)* gene encodes the ortholog CEP-1. CEP-1 is activated by ultraviolet type C (UVC)-induced DNA damage and activates genes that induce germline apoptosis. UVC treatment of gain-of-function *glp-1(ar202gf)/Notch* tumorous animals reduces germline stem cell numbers (and overall tumor size), while UVC treatment of double-mutant *cep-1/p53(gk138);glp-1/Notch(ar202gf)* increases DNA damage adducts and stem cell tumor volume. We compared UVC-induced mitotic stem cell death and animal lifespans for the two different *C. elegans* tumorous strains. *C. elegans* stem cell compartment death has never been observed, and we used engulfed small stem cells, notable by green fluorescent puncta, to count cell death events. We found UVC treatment of *glp-1(ar202gf)* animals increased stem cell death and increased lifespan. However, UVC treatment of double-mutant *cep-1/p53(gk138);glp-1/Notch(ar202gf)* animals decreased stem cell death, increased tumor volume, and decreased animal lifespan. There are pharmacological agents that induce p53-independent cell death of human cells in culture; and two notable protocols are the PARP-trapping agents of temozolomide plus talazoparib and the nucleoside analogue 8-amino-adenosine. It is important to determine ways to rapidly test for pharmacological agents able to induce p53-independent cell death. We tested feeding *cep-1/p53(gk138);glp-1/Notch(ar202gf)* nematodes with either 8-amino-adenosine or temozolomide plus talazoparib and found both were able to decrease tumor volume. This is the first comparison for p53-independent responses in *cep-1/p53(gk138);glp-1/Notch(ar202gf)* animals and showed UVC DNA damage increased tumor volume and decreased lifespan while PARP inhibition decreased tumor volume.

## 1. Introduction

The *p53* gene is conserved from *Caenorhabditis elegans* to humans and is known as the guardian of the genome due to its ability to translate DNA damage signals into signals for cell death and DNA repair even in stem cells [1,2,3,4]. Stress signals as diverse as the activation of an oncogene, or epigenetic modifications, can lead to p53 activation, and this translates into the activation of numerous pathways that can culminate in cell death (apoptosis) and reduced cell proliferation [1,2,3,4]. Loss of wild-type p53 function significantly increases the likelihood of specific types of cancers, and mutations in the *TP53* gene are associated with both sporadic cancers and the familial Li Fraumeni syndrome [1]. The majority of p53 mutations are missense, and many of these cause not only loss of tumor suppressor function but also gained oncogenic activity [5]. As p53 is mutated in over 50% of all human cancers, it is crucial to identify p53-independent pathways to provoke tumor inhibition and tumor cell death. 

Similarly *C. elegans* p53 (CEP-1) activates germ cell apoptosis, and animals with mutation in their *cep-1/*p53 are used as a model organism for examining the influence of drugs on biological pathways in the absence of *cep-1/p53-*induced apoptosis [4,6,7]. *C. elegans* holds potential for pharmacological biomedical research [8,9]. Chemotherapeutic studies focusing on outcomes in *C. elegans* have shown promise for identifying efficacy in treating a number of diseases including cancer [10,11,12,13]. The *C. elegans* germline has been used for screening environmental toxins that activate CEP-1[14]. While CEP-1/p53 promotes apoptosis, it is also required for maintaining genome integrity through normal meiotic chromosome segregation in the germ line [15]. Importantly, the *C. elegans* germ cell genome depends on a number of different DNA repair pathways during proliferation [16]. As such, combining mutations and treating with different pharmacological agents holds promise to find synthetic lethal targets for p53-independent cell death pathways. 

The paradigm in *C. elegans* is that CEP-1/p53 only induces germline cell death when cells are moving from mitosis into meiosis in the transition zone. *C. elegans* gametogenesis provides a model for examining stem cells before they undergo meiosis to become gametes. The proliferating germ cells therefore can be used as a model for cancer stem cells. Germline mitosis occurs in a somatic “niche” that begins on two opposite distal ends, with the meiotic cells developing in the more proximal regions. This germline pattern is controlled by specific proximal and distal somatic gonad signals in a syncytial organization with each nucleus surrounded by a connected cytoplasm that can be disrupted in mutants [17,18]. A schematic of such hermaphrodite gonadogenesis is beautifully represented, along with a clear explanation of the *glp-1(ar202)* Pro phenotype caused by aberrant GLP-1 signaling, in papers from the Hubbard laboratory [17,18]. The region when mitotic cells start to enter prophase of meiosis one can be seen in DAPI-stained gonads, and this region is called the “transition” zone. It is assumed that the germline cell death induced by CEP-1/p53 is only apoptotic and has to be visualized as large transition cells being engulfed by the somatic gonad. All descriptions of germ cell death have suggested that there is no cell death in the mitotic region. As such, no studies to date have examined the influence of loss of CEP-1/p53 function on cell death in the extended mitotic region of Pro phenotype animals with hyper-proliferative germline. CEP-1/p53, the *C. elegans* ortholog to the human p53 protein, functions as a transcription factor that initiates DNA damage-dependent responses by transactivating *egl-1* and *ced-13* (whose orthologs are *PUMA* and *NOXA,* respectively) to promote apoptosis in the presence of DNA damage [6,19,20]. It is highly likely that proliferative mitotic germ cells are capable of signaling DNA damage to CEP-1/p53, as this signaling exists in mammalian stem cells. 

The nematode germline stem cells regulate whole animal lifespan, and a proliferating germline reduces longevity [21,22]. Germline stem cell proliferation is regulated by numerous factors including nutrient sensing [23,24,25,26]. When nematodes express gain-of-function *glp-1 (Notch)*, this promotes germline stem cell proliferation and inhibits differentiation (akin to a germline tumor), which then reduces nematode lifespan. Mutations that increase *C. elegans* lifespan reduce this stem cell proliferation [23,27]. Metabolic stress in the presence of wild-type CEP-1/p53 can reduce nematode lifespan, and in many cases, the loss of normal functioning CEP-1/p53 extends lifespan [28,29,30,31]. Silencing expression of wild-type *cep-1/p53* or the mutant allele *cep-1/p53*(*gk138*) extends the adult nematode lifespan [32]. Therefore, it is possible that the reduction in lifespan seen in gain-of-function *glp-1 (Notch)* animals is due both to the burden of a larger germline size and the metabolic stress signaling to the CEP-1/p53 pathway. If so, then the mutation of *cep-1* would increase the lifespan.

*Glp-1(ar202gf)/Notch* animals are characterized by a proximal tumor phenotype (Pro) that consists of a highly proliferative mitotic region without always presenting as a fully penetrant tumorous germline [17,18]. The allele also has an increased mitotic region due to increased proliferation. We previously reduced the size of the tumor phenotype in *glp-1(ar202gf)/Notch* animals when we treated them with UVC-induced DNA damage. This was likely due to activation of functional CEP-1/p53 because in double-mutant *cep-1/p53(gk138);glp-1/Notch(ar202gf)* animals treated with UVC, there was an expanded size of the proliferative tumorous germline [33]. UVC treatment of *glp-1(ar202gf)/Notch* animals activates CEP-1 target gene pathways, but in mutant *cep-1/p53(gk138);glp-1/Notch(ar202gf)* animals, these pathways are blocked [33]. As such, it was important to follow up our studies to determine how double-mutant *cep-1/p53(gk138);glp-1/Notch(ar202gf)* *C. elegans* were influenced for lifespan and cell death in the proliferative zone. Herein, we report that, in the double-mutant *cep-1/p53(gk138);glp-1/Notch(ar202gf)* animals, the mutated *cep-1*(*gk138*) extends the lifespan of proximal tumor phenotype gain-of-function *glp-1*(*ar202gf*) nematodes. Interestingly, in *cep-1(gk138);glp-1(ar202gf)* double mutants, under conditions where UVC damage increases tumor size, [33] the UVC damage reduced a previously unseen *glp-1(ar202gf)* mitotic germ cell death and reduced nematode lifespan. 

It is critical to identify models and mechanisms to induce p53-independent cell death. We previously identified two pharmacological pathways that are able to induce p53-independent cell death in human cells. These are the nucleoside analogue pathway induced by 8-amino-adenosine and the PARP inhibitor plus DNA damage synthetic lethal pathway induced by temozolomide plus talazoparib [34,35,36,37]. To kill cells in tumors without functional p53, the induction of alternative cell death pathways are needed. PARP inhibitor therapy can function to trap PARP on chromatin and cause synthetic lethal cell death by increasing genomic instability in cells that lack certain DNA repair pathways [38,39,40]. The pharmacological pathways able to induce p53-independent cell death in human cells were herein studied in the context of *C. elegans cep-1(gk138);glp-1(ar202gf)* double mutants. This was done to determine if the *C. elegans* mitotic germ cells could be induced to undergo p53-independent cell death. To our knowledge *C. elegans* germ cell death has only been documented in the transition zone and had never before been documented in the mitotic region. The pharmacological agents tested were the PARP inhibitor talazoparib plus temozolomide and the nucleoside analogue 8-amino-adenosine (previously documented to induce p53-independent cell death in human cells) [34,35]. We observed that mitotic cell death occurred robustly in *glp-1(ar202gf*) animals and was induced pharmacologically but not with UVC in *cep-1(gk138);glp-1(ar202gf)* nematodes. This suggests that *cep-1(gk138);glp-1(ar202gf)* nematodes can be used to screen for compounds that induce p53-independent cell death in a whole animal model. 

## 2. Materials and Methods

### 2.1. Growth Media and C. elegans Maintenance

All strains were grown on nematode growth media (NGM) and were grown at either 15 °C, 20 °C, or 25 °C. Further, 1 L of NGM includes: 3 g NaCl (Fisher), 17 g agar (Fisher), 2.5 g peptone (Becton, Dickinson and Company), and 1 mL cholesterol (5 mg/mL in 95% ethanol). We sterilized by autoclaving and added 1 mL of 1M CaCl_2_, 1 mL of 1M MgSO_4_, and 25 mL 1M potassium phosphate, pH 6. The plates were dried, and 200 µL OP50 *E. coli* in LB was pipetted onto the center of the plate for the worms to consume. M9 Buffer was prepared using 3 g KH_2_PO_4_, 6 g Na_2_HPO_4_, 5 g NaCl, and 1 mL 1M MgSO_4_, followed by adding H_2_O up to 1 L. For drug treatments, concentrated (4×) heat-killed OP50 (HKOP50) was prepared by inoculating a single OP50 colony in 200 mL LB broth and was grown overnight in a 37 °C shaker. OP50 was transferred to a 65 °C water bath for 30 minutes, followed by making 40 mL aliquots and centrifuging at 6000 rpm. The supernatant was discarded, and the pellet was resuspended in 10 ml fresh LB and stored at 4 °C.

### 2.2. C. elegans Strains

The following mutant strains were used: *cep-1(gk138)* *I bcls39[P(lim-7)ced-1::GFP + lin-15(+)]* (JBC1), *cep-1(gk138) I glp-1(ar202gf) III* (JBC2), *I glp-1(ar202gf) III bcls39[P(lim-7)ced-1::GFP + lin-15(+)] V* (JCB6), and *cep-1(gk138) I glp-1(ar202gf) III bcls39[P(lim-7)ced-1::GFP + lin-15(+)] V* (JBC7). The *cep-1(gk138) I glp-1(ar202gf) III bcls39[P(lim-7)ced-1::GFP + lin-15(+)] V* (JBC7) strain was constructed by the Bargonetti Laboratory: *cep-1(gk138) I bcls39[P(lim-7)ced-1::GFP + lin-15(+)] V* (JBC1) strain males were crossed with *cep-1(gk138) I glp-1(ar202gf) III* (JBC2) strain hermaphrodites, yielding an F1 population. F1 progeny (F2 generation) laid at 15 °C of worms were selected if F2 worms laid at 25 °C developed full-body tumors, resulting in sterility. The selection process was repeated with the offspring of the selected F2 worms at 15 °C, and epifluorescence microscopy was used to identify a brood in which 100% of worms screened tested positive for germline GFP expression. The strain *bcIs39[P(lim-7)ced-1::GFP+lin-15(+)]* (MD701) were also used [41].

### 2.3. Lifespan Assay

Adult worms were picked from the stock plate and transferred onto a freshly seeded NGM plate to lay eggs for 2 h at either 15 °C or 20 °C (for animals to be shifted to 25 °C). Worms were removed from the plate leaving only the laid eggs. The plates were then incubated at 15 °C or 25 °C accordingly and allowed to grow until they reached L4 larval stage. Non-L4s were removed from the plate, and the synchronized worms were grown for an additional 24 h. Worms were then monitored and scored every other day. Worms were transferred to newly seeded plates as necessary to avoid mixing with new progeny and starvation via OP50 depletion. A worm pick was used to detect movement in response to touch on its body to determine status of worms. Deaths were observed and scored as events for each worm. Worms that died through embryonic matricide (bagging), burrowing in the agar, or desiccation along the edge of the plate were censored. 

### 2.4. UVC Treatments

UVC treatments were done with a Spectrolinker. Worms were picked and transferred to NGM plates without bacteria. Worms were then exposed to 50 J/m^2^ UVC or left untreated as controls. Worms were then placed back into seeded NGM plates and allowed to recover. For lifespan experiments, UVC treatments were done 24 h after the L4 stage. For cell death experiments, worms were placed back onto seeded NGM plates to recover for 24 h, followed by live imaging.

### 2.5. Cell Death Analysis and Scoring

Analysis of germline cell death events were done using the CED-1::GFP reporter. Worm slides were prepared 24 h after recovery from either UVC or drug treatments (and untreated controls). Agar pads were prepared by adding 55 µL to 60 µL of molten 2% agarose, flattening by another slide, and adding 5 µL 3 mM levamisole dissolved in M9 buffer to anesthetize the worms. Worms were picked from seeded NGM plates and placed in the agar pad with levamisole. Normarski (DIC) and green fluorescent images were taken using the Nikon Eclipse Ti-S Fluorescent Microscope at 20× objectives. Images were processed, and cell death events were scored using ImageJ with Bio-Formats plug-in. 

### 2.6. Drug Treatment Assay

#### 2.6.1. 8-Amino-Adenosine Treatment

Worms were grown on heat-killed OP50-seeded NGM plates at 15 °C following a 3-hour egg lay. Bacterial lawn solutions for the three experimental groups were prepared using 1:3 dilutions: (1) H_2_O (control): 50 μL autoclaved water + 150 μL 4× concentrated heat-killed OP50 *E. coli*; (2) 5 μM (experimental): 50 μL 5μM 8AA + 150 μL of 4× concentrated heat-killed OP50 bacteria; and (3) 50 μM (experimental): 50 μl 50 μM 8AA + 150 μL 4× concentrated heat-killed OP50 bacteria. Worms were shifted to the tumor-inducing temperature of 25 °C at the L4 stage and DAPI stained 24 h later. Confocal images of each germline bend were taken at 20× objectives of worms mounted on 3% agar pad slides.

#### 2.6.2. Temozolomide and Talazoparib Treatment

The following solutions were prepared for the drug treatment: (1) 50 µL of 1% dimethyl sulfoxide (DMSO, in M9) + 150 µL 4× concentrated HKOP50 (vehicle control) and (2) 50 µL temozolomide (1 mM) and talazoparib (10 µM) + 150 µL 4× concentrated HKOP50 (both drugs in 1% DMSO). Adult worms were transferred onto empty NGM plates to rid the live OP50 from their bodies. Worms were transferred to plates with heat-killed OP50 containing vehicle control or the drugs and allowed to lay eggs for 2 h, followed by removal of worms and incubation of plates with eggs at 15 °C until L4 stage. Then, worms continued to grow for 24 h at 25 °C to induce tumor growth. Worms were then removed from plates and prepared for imaging.

### 2.7. Measuring Proliferative Capacity 

Worms were collected and transferred into an Eppendorf tube with 1 mL M9 buffer. Worms were centrifuged (1000 rpm) for 1 min, and the supernatant was discarded. The pellet was fixed for 5 min under 1 mL 95% ethanol, followed by another centrifugation for 1 min at 1000 rpm. The supernatant was removed, and 1 drop of mounting media with DAPI (Vectashield) was added to the pellet and wrapped in aluminum foil and stored at 4 °C for 30 min up to one day before imaging. Worms were imaged by adding 5 µL of mounting media with DAPI solution containing fixed worms onto 2% agarose pads on glass slides. Normarski (DIC) and DAPI (blue fluorescent) images were taken in multiple z-stacks of the germline using Nikon A1 Confocal Laser Scanning Microscope at 20× objectives. Image acquisition and analysis of the germline was performed using NIS Elements.

### 2.8. Statistical Analysis

GraphPad Prism was used to input and analyze all data. Strength of statistical evidence was computed using the unpaired two-tailed Student’s *t*-test for cell death and proliferative capacity data. Kaplan–Meier survival curves were generated for each pooled lifespan experiments presented in the figures. The log-rank test was used for statistical analysis of survival curves. The *p*-values are presented in the figure legends.

## 3. Results

### 3.1. The cep-1(gk138) Loss-of-Function Mutation Extends the Lifespan of Tumorous Gain-of-Function glp-1(ar202gf)/Notch C. elegans

CEP-1 induces germ cell apoptosis and promotes DNA repair in response to stress, including stress following UVC-induced DNA damage [4,6,7,33]. In addition, the activation of CEP-1 reduces *C. elegans* lifespan [29,30,31,32]. To examine the influence of CEP-1 on the lifespan of *glp-1*(*ar202gf*) proliferative germline animals, we compared *glp-1(ar202gf*)/*Notch* germline Pro tumor mutants to *cep-1*(*gk138*);*glp-1*(*ar202gf*) double mutants. When eggs are shifted to 25 °C for worm development, the germ-line tumor formation is initiated by the gain-of-function mutant *glp-1(ar202gf*) (that have a missense base-pair change at G529E in the LIN-12 Notch repeats) [18,26,42]. The mutant GLP-1 protein assumes an abnormal conformation at 25 °C, remaining constitutively active to promote mitotic division independently of the LAG-2 ligand that is normally required for GLP-1/Notch activation [43]. To compare CEP-1*-*independent and CEP-1-dependent cell death in this *C. elegans* germline mutant background, we constructed the strains *cep-1(gk138); glp-1(ar202); bcls39[P(lim-7)ced-1::GFP + lin-15(+)]* and *glp-1(ar202); bcls39[P(lim-7)ced-1::GFP + lin-15(+)]*. These are the animals used throughout these studies.

These strains were created to establish a model in which lifespan and tumor development could be monitored while also monitoring cell death by using the CED-1::GFP fusion protein to mark dying cells. The CED1::GFP is a strong marker for cell death and can be scored for cell death that is both apoptotic and necrotic [44,45]. We assumed that cell death in the mitotic region would be hard to see, as the proliferative cells are very small. Moreover, it was important to score for all types of cell death because previous literature has suggested that no apoptotic cell death occurs in mitotic germ cells. Therefore, the cell death scored by green puncta in the mitotic region could be a combination of many different varieties of cell death. The goal of this work was to determine if cell death occurred in the mitotic proliferative region and if DNA damage could increase the amount of such cell deaths. 

**Figure 1:**
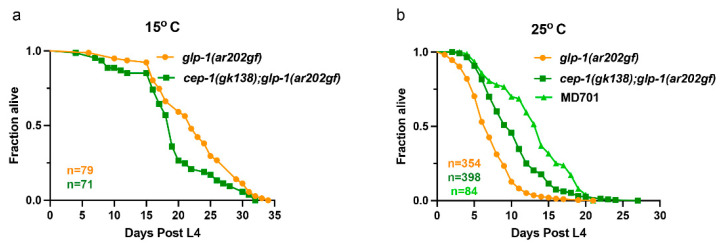
Loss of wild-type *cep-1* led to a rescue of the short-lived lifespan in worms with germline tumors. (**a**) At the permissive temperature (15 °C), *glp-1(ar202gf)* animals had a slight increase in lifespan (median = 22 days) compared to the *cep-1(gk138); glp-1(ar202gf)* animals (median = 19 days), in the absence of a germline tumor (*p* = 0.0080). (**b**) At the restrictive temperature (25 °C), both worm strains yielded a Pro tumor germline phenotype. Loss of wild-type *cep-1* in the *cep-1(gk138); glp-1(ar202gf)* animals resulted in an increased lifespan (median = 10 days) compared to *glp-1(ar202gf)* animals (median = 7 days) in the presence of a tumor (*p* < 10^−15^), but both were less than the MD701 (wild-type) animals (*p*-value = 2.8 × 10^−5^). MD701 animals had a median lifespan of 14 days. Survival curves represent data pooled from multiple biological replicates. The *n* values represent the total number of worms in the pooled data set. *p*-values for comparing two groups were obtained using the log-rank (Mantel–Cox) statistical test.

The *glp-1(ar202*gf*)* genotype causes animals maintained at 25 °C to have a shorter lifespan [46]. The *glp-1(ar202*gf*)* lifespan is extended by the RNAi-mediated knockdown of *mre-11* plus gamma irradiation because inhibiting homologous recombination increases radiation sensitivity and tumor reduction in the distal region of the worm germline [46]. We performed lifespan assays on both the *glp-1(ar202*gf*)* and the *cep-1(gk138);glp-1(ar202*gf*)* double mutants at both 15 °C (without a Pro tumor germline phenotype) and at 25 °C (with a Pro tumor germline phenotype). The experiments were designed to maximize the Pro tumor phenotype [18]. This included having the animals lay eggs at 20 °C for the 25 °C shift experiments. All animals lived longer at 15 °C (without a Pro tumor germline) than they did at 25 °C (with a Pro tumor germline) (Figure 1). However, we observed an extension of lifespan of the *cep-1(gk138);glp-1(ar202*gf*)* animals compared to the *glp-1(ar202*gf*)* animals at 25 °C though not at 15 °C. (Figure 1b and 1a, respectively). At the tumor-inducing temperature of 25 °C, both the *glp-1(ar202*gf*)* and the *cep-1(gk138);glp-1(ar202*gf*)* animals had highly penetrant Pro tumor germline phenotypes. 

We observed that compared to the *glp-1(ar202*gf*)* at 25 °C, the *cep-1(gk138);glp-1(ar202*gf) had an overall increased adult lifespan (Figure 1b). This was still less than the survival duration for the control MD701 animals without tumors (*p*-value = 2.8 × 10^−5^). At 25 °C, the median survival time for the *glp-1(ar202*gf*)* animals was 7 days, and the median survival for the *cep-1(gk138);glp-1(ar202*gf*)* animals was 10 days (while the MD701 animals had a median of 14 days). This recapitulated data show that in the presence of metabolic stress, the loss of normal functioning CEP-1 can extend lifespan [28,29,30,31]. Here, we posit that having a Pro tumor phenotype causes metabolic stress. If we consider having a tumorous germline a stress, then it appears this stress activates whole animal *cep-1* to reduce lifespan. Therefore, the *cep-1(gk138);glp-1(ar202*gf*)* animals show a rescue of the short-lived phenotype in the *glp-1(ar202*gf*)* germline tumor animals.

### 3.2. UVC Treatment Reduced Survival of cep-1(gk138);glp-1(ar202gf) Animals

**Figure 2:**
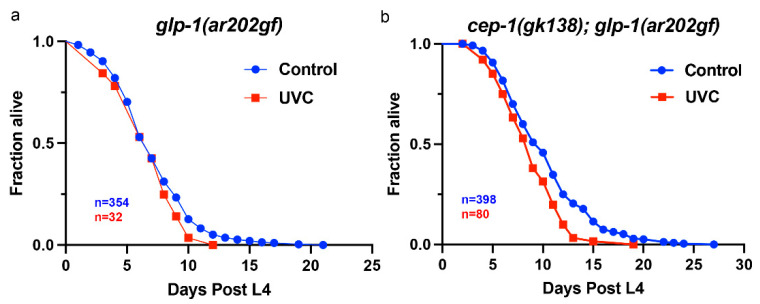
UVC treatment of tumorous worms affected their overall survival. Worms were treated with 50 J/m^2^ UVC 1 day post L4 stage. (**a**) Inducing DNA damage using UVC treatment on tumorous *glp-1(ar202gf)* worms led to no change in survival from the untreated counterpart (median = 5 days). (**b**) UVC treatment of tumorous *cep-1(gk138); glp-1(ar202gf)* animals, however, led to reduced survival overall (median = 9 days) compared to their control group (median = 10 days, *p* = 0.0018). Survival curves represent data pooled from multiple biological replicates. The *n* values represent the total number of worms in the data set. *p*-values were obtained using the log-rank test (Mantel–Cox).

Proliferating germ cells reduce longevity [21,22], which suggested that *cep-1(gk138);glp-1(ar202gf)* animals with a UVC-induced increase in tumor size would have a shorter lifespan. We carried out lifespan assays for both *glp-1(ar202gf)* and *cep-1(gk138);glp-1(ar202gf)* animals and treated these worms with 50 J/m^2^ UVC one day after the worms were at L4 stage. The dosage of 50 J/m^2^ UVC was based on our previous publication and was used for all experiments [33]. The lifespan of *glp-1(ar202gf)* animals was unchanged following UVC treatment, while the lifespan of *cep-1(gk138);glp-1(ar202*gf*)* double-mutant animals was reduced (Figure 2). The median survival time of the UVC-treated *glp-1(ar202gf)* animals and the control untreated animals was 5 days (Figure 2a). The UVC-treated *cep-1(gk138);glp-1(ar202gf)* animals’ median survival time was 9 days compared to the untreated median survival of 10 days (*p*-value = 0.0018)(Figure 2b). These results showed that inducing DNA damage decreased the lifespan of tumorous animals in the absence of functional CEP-1/p53. These data correlated with the fact that UVC damage increases the tumor size of *cep-1(gk138);glp-1(ar202gf)* double mutants [33]. 

### 3.3. UVC-Induced DNA Damage Reduced Mitotic Cell Death in cep-1(gk138);glp-1(ar202) Worms

We previously reported that UVC-induced DNA damage of the *glp-1(ar202*gf*)* animals reduces tumor size, but in double-mutant *cep-1(gk138);glp-1(ar202gf)* animals, the UVC treatment induces unrepaired DNA lesions, increases germline cell proliferation, and increases tumor size [33]. We predicted that UVC was able to induce CEP-1 provoked mitotic cell death in *glp-1(ar202gf)* animals but not in the *cep-1(gk138);glp-1(ar202gf)* counterpart. The strains *cep-1(gk138); glp-1(ar202gf); bcls39[P(lim-7)ced-1::GFP + lin-15(+)]* and *glp-1(ar202gf); bcls39[P(lim-7)ced-1::GFP + lin-15(+)]* were monitored for CED-1::GFP fusion-labeled protein dying cells. Imaging for the CED-1::GFP fusion protein is a sensitive method for visualizing the somatic sheath cell that encircles early apoptotic corpses in the transition zone of the germline during engulfment [47]. However, the cells in the proliferative zone are very small. As such, we reasoned proliferative cells would be much smaller green spots and would not appear as empty engulfed corpses. We fixed animals to make sure that the smaller green cells/puncta in the mitotic region always overlapped with DAPI-stained DNA. Using this method, we quantified deaths by counting GFP puncta in the hyper-proliferative germline. We carried out live imaging on both strains 24 h after UVC treatment. By using the CED-1::GFP reporter to score the number of cell death events, we determined the change in the frequency of cell death events. As described in the introduction, CED1::GFP is a strong marker for cell death and will score for cell death that is both apoptotic and necrotic [44,45]. 

**Figure 3:**
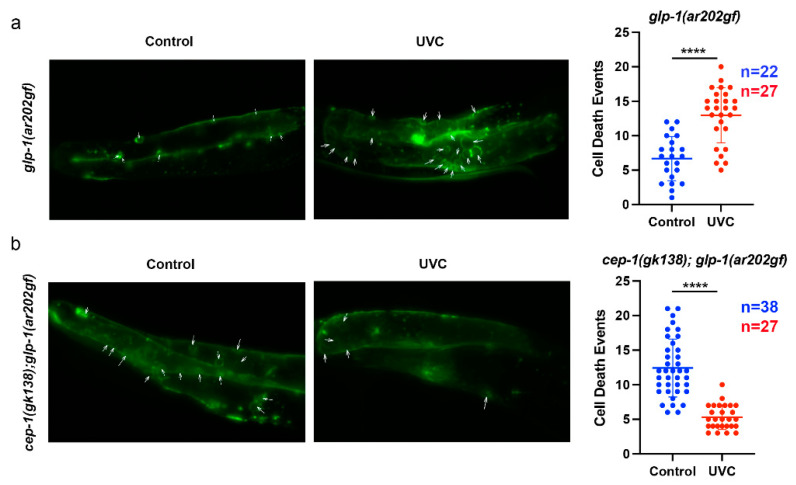
UVC treatment of *glp-1(ar202gf) and cep-1(gk138); glp-1(ar202gf)* animals led to changes in mitotic cell death events. Representative green fluorescent images of live worm germline shown as maximum intensity projections (200× magnification). Cell death events were detected using the CED-1::GFP reporter, observed as green circles/puncta (white arrows), and scored as cell death events. (**a**) UVC treatment of *glp-1(ar202gf)* animals with wild-type *cep-1* increased mitotic cell death. The average number of these cell death events 24 hours after UVC treatment is greater (mean = 12.96 ± 4.005) compared to the control group (mean = 6.682 ± 3.213, *p* = 3.14 × 10^−7^). (**b**) UVC treatment of *cep-1(gk138); glp-1(ar202gf)* animals led to decreased mitotic cell death events. The average number of cell death events is lower under UVC treatment (mean = 5.296 ± 1.772) compared to the control group (mean = 12.42 ± 4.202, *p* = 1.12 × 10^−11^). The *n* values for each scatter dot plot (in (**a**,**b**)) represent the number worms used for scoring (one gonad arm per worm). Data shown (in (**a**,**b**)) are represented as mean ± SD. *p*-values were obtained using an unpaired two-tailed Student’s *t*-test. The following format was used to assign significance, with “****” used for a *p*-value of less than 0.0001.

UVC treatment increased the mitotic cell death events in *glp-1(ar202gf)* animals from an average of 6.68 events to an average of 12.96 cell death events per gonad arm per worm (*p*-value = 3.14 × 10^−7^) (Figure 3a). In contrast, UVC treatment decreased the mitotic cell death events in *cep-1(gk138); glp-1(ar202gf)* animals (Figure 3b). Interestingly, we observed a high average number of cell death events in the *cep-1(gk138);glp-1(ar202gf)* animals prior to UVC treatment (12 events per gonad arm). This may be related to the synthetic lethality posed by loss of CEP-1 function causing reduced double-strand breaks (as needed for proper homologous recombination during mitosis) and increased genomic instability [48,49]. Here, we show that inducing DNA damage with UVC reduces mitotic proliferative germ cell death frequency. The average cell death events for the UVC *cep-1(gk138); glp-1(ar202gf)* animals was 5.29 events compared to the untreated group that had an average of 12.42 events per gonad arm (*p* = 1.12 × 10^−11^). This increased basal cell death in the absence of functional CEP-1 was surprising, as CEP-1 is usually considered as an inducer of apoptosis and cell cycle arrest [20]. However, our data after UVC treatment suggest that the role for CEP-1 in the mitotic germ cells may be involved more directly in the maintenance of genomic stability through promoting homologous recombination for reproductive health [48]. It is not yet clear if the decrease in dead cells following UVC treatment in *cep-1(gk138); glp-1(ar202gf)* animals helps to promote recombination and is seen more in the mitotic region or the transition zone. This will be followed up in a future study, as there are most likely many factors responsible for the changes we observed in *cep-1(gk138); glp-1(ar202gf)* animals. Thus, future studies will focus on earlier time points during development and analysis of images at higher magnification. 

### 3.4. Drugs That Induce Human p53-Independent Cell Death Decreased the Number of Cells in the cep-1(gk138);glp-1(ar202gf) Tumorous Germline

**Figure 4:**
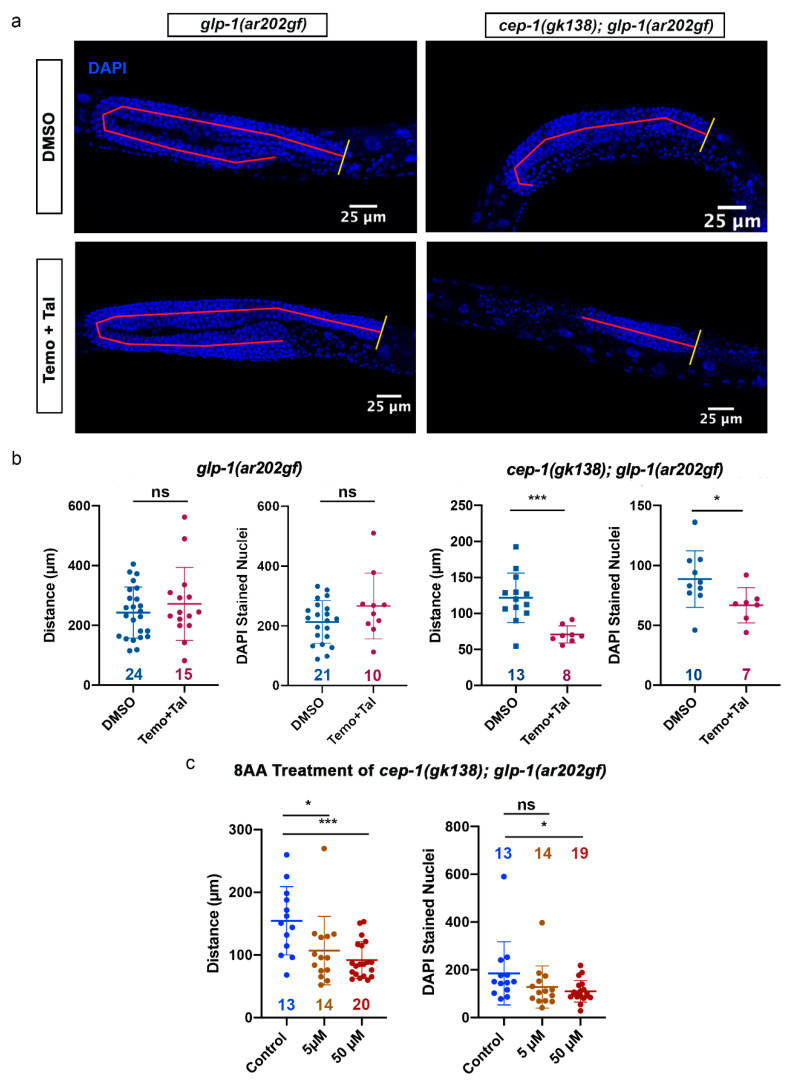
Treatment of tumorous worms with chemotherapeutic agents showed changes in proliferative capacity in the absence of wild-type *cep-1*. (**a**) Representative maximum intensity projection images of the *C. elegans* germline for *glp-1(ar202gf)* (left column) and *cep-1(gk138); glp-1(ar202gf)* animals (right column) treated with dimethyl sulfoxide (DMSO) or temozolomide plus talazoparib (Temo+Tal). Distance from the distal end of the germline (yellow line) to the mitotic region/transition zone boundary (MR/TZ) was drawn and measured using NIS Elements (red line). Nuclei were counterstained with DAPI. (**b**) Quantitative analysis for proliferative capacity of *glp-1(ar202gf)* and *cep-1(gk138); glp-1(ar202gf)* animals under combination treatment Temo+Tal. Treatment of *glp-1(ar202gf)* animals with Temo and Tal had no effect on distance between the distal end and the MR/TZ boundary: neither average distances of 242.8 ± 85.45 µm for the control and 271.9 ± 122.1 µm for the treatment group (*p* = 0.3858) nor the amount of DAPI-stained germ cell nuclei scored: 212 ± 71.31 under DMSO and 266 ± 109.8 under Temo+Tal (*p* = 0.1122). The *cep-1(gk138); glp-1(ar202gf)* animals had a decrease in both the distance (121.8 ± 34.33 µm in the control group and 71.01 ± 11.90 µm in the treatment group (*p* = 0.0008)) and a fair reduction in the amount of mitotic germline cells (88.7 ± 23.6) in the control group and 66.86 ± 14.75 in the treatment group (*p* = 0.0474)) under Temo+Tal treatment. (**c**) Treatment of *cep-1(gk138); glp-1(ar202gf)* double-mutant animals with 8AA led to reduction in the DTC-to-MR/TZ distance, with a greater reduction at a concentration of 50 µM: an average of 154.6 ± 54.48 µm in the control group to an average of 91.95 ± 29.36 µm in the treatment group (*p* = 0.0002). Treatment at 50 µM reduced the number of mitotic germline cells from an average of 185.3 ± 132.5 in the control group to 110.4 ± 45.48 in the treatment group (*p* = 0.0292). The *n* values for each scatter dot plot (in b and c) represent the number of gonad arms scored. Data shown (in b and c) are represented as mean ± SD. *p*-values were obtained using an unpaired two-tailed Student’s *t*-test. The following format was used to assign significance, with “ns” indicating no significance, “*” indicating a *p*-value of less than 0.05, and “***” indicating a *p*-value of less than 0.001.

The fact the p53 is mutated in over 50% of all cancers makes using models with loss of functional p53 a good surrogate examining p53-independent outcomes. We see that the constructed strains of *cep-1(gk138); glp-1(ar202gf); bcls39[P(lim-7)ced-1::GFP + lin-15(+)]* and *glp-1(ar202gf); bcls39[P(lim-7)ced-1::GFP + lin-15(+)]* allow for the comparison of complex outcomes that influence tumor size in the presence or absence of CEP-1/p53 signaling. We reasoned that comparison of these two strains would enable the evaluation of chemotherapeutic protocols found in preclinical trials to activate p53-independent cell death pathways [34,35]. 8-amino-adenosine (8AA), a ribose sugar nucleoside analogue, and the poly-ADP-ribose inhibitor (PARPi) talazoparib in combination with temozolomide activate p53-independent cell death in triple-negative breast cancer cells [34,35]. The mitotic proliferative zone of the *C. elegans* germline contains only stem cells. These stem cells have been shown to be actively replicating and easily quantitated for analysis of the proliferative population [50]. We used this proliferative germline assessment method as a fine-tuned way to address the influence of chemotherapeutic drugs on proliferation in a whole animal Pro tumor phenotype stem cell population. This method was recently used to show that BEC-1 acts non-cell-autonomously to promote the proliferation of germline stem cells [51]. We wanted to know if chemotherapeutic drug treatment of *C. elegans* could do the opposite and reduce germline stem cell proliferation. We also wanted to determine if the chemotherapeutic drugs would inhibit stem cell proliferation independently of p53/CEP-1. The two aforementioned strains were compared for treatment with 8-amino-adenosine (8AA) and the PARPi talazoparib in combination with temozolomide (Figure 4). We tested the outcome of treatment with 8-amino-adenosine (8AA) or the PARPi talazoparib in combination with temozolomide on stem cell proliferation in nematodes shifted to the GLP-1gf/Notch proliferation promoting conditions when they reached adulthood. As such, all experiments to examine influences on the proliferative zone in the drug treatments were carried out on animals shifted to 25 °C at the L4 stage. This allowed us to examine the increased mitotic region due to increased proliferation in the absence of the Pro tumor phenotype. 

We used the previously described different genotypes to investigate effects of CEP-1/p53 on proliferative capacity and measured the length from the distal tip cell (DTC) to the mitotic region/transition zone (MR/TZ) boundary to indicate the length of the proliferative region and also counted DAPI-stained nuclei in these mitotic regions for further quantification [26,51]. Proliferation of cells in the *C. elegans* germline progress from the distal end bordering the distal niche containing the distal tip cell (DTC) to the transition zone, where cells assume a crescent-shaped morphology characteristic of early meiotic prophase [26,51]. We carried out fixation of the worms and DAPI staining of the DNA in whole worms in order to analyze the germ cell content of the *C. elegans* germline under vehicle control and treatment with talazoparib with temozolomide (Temo+Tal) (Figure 4a). Experiments to determine the induction of cell death and the influence of these drugs on lifespan will be important experiments to carry out in the future.

Talazoparib plus temozolomide treatment of *glp-1(ar202gf)* animals resulted in no demonstrable change in either the length from the distal tip cell to the mitotic region/transition zone (DTC-to-MR/TZ) boundary or the number of germ cell nuclei (Figure 4b, left panel). The average distance in *glp-1(ar202gf)* animals for both the vehicle control and the talazoparib with temozolomide were 242.8 µm and 271.9 µm, respectively (*p* = 0.3858). Importantly, there was a reduction in both the distance and the number of germ cell nuclei in the double-mutant *cep-1(gk138); glp-1(ar202gf)* animals (Figure 4b, right panel). The average distance in *cep-1(gk138); glp-1(ar202gf)* animals with talazoparib plus temozolomide treatment was 71.01 µm compared to the control group, which had an average distance of 121.8 µm (*p* = 0.0008). The number of germ cells was reduced from 88.7 in the control group to 66.86 with the drug treatment.

We also treated the *cep-1(gk138); glp-1(ar202gf)* animals with 8-amino-adenosine to determine if the ribose sugar nucleoside analogue was capable of reducing the proliferative capacity of tumorous worms with *cep-1(gk138)* loss-of-function mutation (Figure 4c). The 8-amino-adenosine treatment reduced both the length from the distal tip cell to mitotic region/transition zone (DTC-to-MR/TZ) and the number of germ cells in the *cep-1(gk138); glp-1(ar202gf)* double-mutant animals. A concentration dependence was observed, with the average distance reduced from 107.0 µm with the lower concentration to an average of 91.95 µm (*p* = 0.0002) with the higher-concentration treatment (Figure 4c, left panel). The higher-concentration 8-amino-adenosine treatment also reduced the average number of germ cells per gonad arm from 185 to 110 germ cells per gonad arm (*p* = 0.0292) (Figure 4c, right panel). 

## 4. Discussion

**Figure 5:**
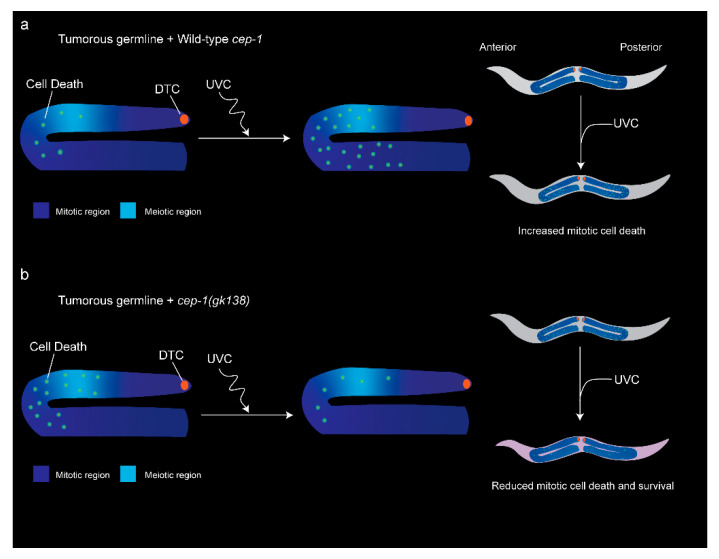
Hypothetical model for cell death outcomes in any tumorous *C. elegans* gonad with or without functional CEP-1 under UVC treatment and outcomes on overall worm survival. Schematic of lateral cross-section view of adult anterior gonad arm shown on the left and a longitudinal section view of the whole hermaphrodite on the right. The *glp-1(ar202gf)* mutation causes the resulting GLP-1/Notch receptor to be constitutively active at 25 °C, allowing germ cells to undergo mitosis without direct interaction of the ligand, LAG-2, where it is highly concentrated at the distal tip cell (DTC, orange). A proximal tumor phenotype (Pro) in the somatic gonad is indicated by an increased mitotic region (dark blue). Variability of the Pro phenotype were observed where the transition zone and meiotic regions (shown as light blue) were variable (see Figure 4a). (**a**) In the presence of wild-type *cep-1* (top panel), UVC induces CEP-1/p53-dependent mitotic cell death (green dots). (**b**) In a loss-of-function *cep-1(gk138)* mutation (bottom panel), UVC reduces the overall starting mitotic cell death events. This allows for mitotic germline proliferation to increase and resulted in a larger tumorous germline and reduced overall worm survival (the light-violet worm indicates less cell death events resulting in reduced overall survival).

The p53 protein is clearly a major deterrent to cancer formation in many organisms. Studying the influence of CEP-1/p53 on tumorigenesis in the easily tractable *C. elegans* requires examining the germline, as the other cells in the adult animal do not proliferate. In order to evaluate the contribution of CEP-1 function in a *C. elegans* tumor model, we compared single-mutant *glp-1(ar202gf);bcls39[P(lim-7)ced-1::GFP + lin-15(+)]* worms to double-mutant *cep-1(gk138);glp-1(ar202gf);bcls39[P(lim-7)ced-1::GFP + lin-15(+)]* worms. Interestingly, we observed an increased lifespan in *cep-1; glp-1* double-mutant animals at 25 °C but not at 15 °C. This follows a previous observation that metabolic stress in the presence of wild-type CEP-1/p53 can reduce nematode lifespan, and the loss of normal functioning CEP-1/p53 extends lifespan [28,29,30,31]. Under the germline tumor stress conditions, the loss of normal-functioning CEP-1/p53 wins over the gain-of-function *glp-1 (Notch)* to result in increased nematode lifespan [23,27]. We have reported here, for the first time, that UVC irradiation increased cell death in single-mutant *glp-1(ar202gf);bcls39[P(lim-7)ced-1::GFP + lin-15(+)]* worms (Figure 3a and Figure 5). In contrast, after UVC irradiation, double-mutant *cep-1(gk138);glp-1(ar202gf);bcls39[P(lim-7)ced-1::GFP + lin-15(+)]* worms had reduced cell death (Figure 3b and Figure 5). As we hypothesized, UVC treatment increased germ cell death in the tumorous germline of single-mutant animals (Figure 2a, Figure 3a and Figure 5). Interestingly, UVC treatment decreased cell death in the double-mutant non-functional CEP-1/p53 animals, and this correlated with a decrease in lifespan (Figure 2b, Figure 3b and Figure 5).

CEP-1/p53 is important for meiotic fidelity. In *C. elegans*, different CEP-1/p53 mutants that are transcriptionally inactive are characterized by a separation of function in maintaining genomic fidelity, with *cep-1/p53*(*gk138*) remaining more viable than *cep-1/ p53(*lg12501) [48]. The synthetic lethality posed by mutated CEP-1/p53 loss-of-function is due to reduced double-strand breaks [48,49]. It will be interesting in the future to determine if the separation of function of *cep-1/p53* allele lg12501 or feeding animals with *cep-1* RNAi will produce the same outcomes that we have documented here with the *cep-1* allele of *cep-1/p53*(*gk138*). 

We previously documented that UVC-induced nuclear DNA damage is not effectively repaired in *cep-1(gk138)* animals [33]. Thus, UVC damage *cep-1(gk138); glp-1(ar202gf)* animals may result in increased DNA double-strand breaks during DNA replication, which then promote recombination that allows for cell survival. In the absence of DNA damage, mutation of CEP-1/p53 causes reduced double-strand breaks [48,49]. This may be why more cell death is observed in *cep-1(gk138)* animals before UVC treatment. The increased germ cell deaths would be a result of increased replication stress when there are no UVC-induced DNA breaks. The replication stress would be reduced by the DNA breaks that happen after UVC or chemotherapeutic treatment (Figure 3b and Figure 5b). Interestingly, the *glp-1(ar202gf)* had a longer proliferative zone than *cep-1;glp-1* (248 vs. 121 µm). We propose that this difference in length is due to the *cep-1(gk138); glp-1(ar202gf)* animals having increased mitotic cell death (shown in Figure 3). This could be the result of less homologous recombination that results in mitotic catastrophe, driving cell death.

**Figure 6:**
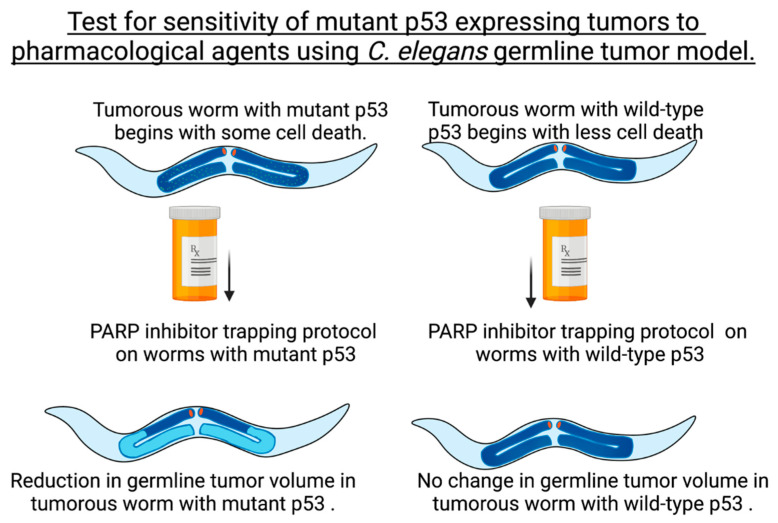
Targeted treatment with a PARP inhibitor protocol reduced germline tumor volume in animals with mutant but not wild-type CEP-1/p53. The figure shows longitudinal section views of the whole hermaphrodite. The increased mitotic region is shown in dark blue for the gonad arms, and lighter blue then represents meiotic regions of the gonad arms on the bottom left. Data from Figure 3 and Figure 4 suggest that *C. elegans* tumorous germlines can be compared in response to pharmacological agents to predict which compounds are best for reducing tumor volume and inducing cell death in mutant CEP-1/p53 backgrounds (as seen for the bottom left hermaphrodite). Treatment of *C. elegans* with a PARP inhibitor protocol (using temozolomide plus talazoparib) or the p53-independent death-inducing nucleoside analogue 8-amino-adenosine reduced germline tumor volume in animals with mutant CEP-1/p53 (as seen for the bottom left hermaphrodite). Created with BioRender.com.

Treatment of worms with either temozolomide plus talazoparib or 8-amino-adenosine decreased the proliferative capacity of nuclei in the mitotic region of the germline in a *cep-1/p53-*independent manner. Both these treatments significantly reduced the length of the proliferative zone and also decreased the number of mitotic cells in the double mutant animals. The same pattern was not noted in single-mutant animals with functional CEP-1. We did not carry out experiments on the *cep-1(gk138); glp-1(ar202gf)* double-mutant animals with DNA-damaging chemotherapeutics that induce a p53 response because we were interested in identifying alternative mechanisms besides p53 pathways to induce cell death. Our previous data testing a DNA damage-inducing chemotherapeutic on *cep-1* mutant animals showed that more DNA damage accumulates in the absence of functional p53, and thus, we anticipate that standard DNA-damaging chemotherapeutics would mimic the response seen with UVC. UVC causes the tumor size to increase [33]. However, treatment with pharmacological p53-independent cell death inducers causes *C. elegans* tumorous germlines with mutant p53 to reduce in size (Figure 6). 

## 5. Conclusions 

We conclude that we have constructed a *cep-1(gk138); glp-1(ar202gf)* double-mutant strain (*cep-1(gk138) I glp-1(ar202gf) III bcls39[P(lim-7)ced-1::GFP + lin-15(+)] V)* that is useful for detection of pharmacologically induced p53-independent cell death in mitotic germ cells. Furthermore, this strain is useful for assessing the ability of pharmacological agents to reduce *C. elegans* germline tumor size. We conclude that studying the *C. elegans* germline tumor model with and without functional p53 enables the influence of whole animal cell stress to be assessed. The reduction in lifespan seen in gain-of-function *glp-1 (Notch)* animals might be due both to the burden of a large germline size as well as the metabolic stress signaling that comes from activation of the CEP-1/p53 pathway. A recent report delves in to the complexity of p53 and aging, showing that autophagy suppresses the p53 pathway to reduce the pace of aging [52,53]. We propose that inducing p53-independent cell death pathways will not only be good for cancers with mutant p53 but will also reduce the deleterious influences often seen by treating people with DNA-damaging agents that activate the p53 pathway. Chemical inhibition of PARP in *C. elegans* reduces axonal degeneration, which coordinates with the lack of a change in tumor volume in the presence of a functional CEP-1/p53 pathway [54,55]. It remains to be determined how the PARP inhibitor protocol resulted in decreased tumor volume in the absence of the CEP-1/p53 pathway. All this and more can be tested in the future using the amazing model system of germline tumorous *C. elegans*.

## Data Availability

Data presented in this study are maintained within this article.
